# Supplementary material for: Crosstalk between nitric oxide and retinoic acid pathways is essential for amphioxus pharynx development
Source: eLife. 2021 Aug 25;10:e58295. doi: 10.7554/eLife.58295 (PMC8387019; doi:10.7554/eLife.58295)
Supplement: Supplementary file 1. [file elife-58295-supp1.docx]

| **Primers for *in situ* hybridization probes** | | | |
| --- | --- | --- | --- |
| **Gene** | **Forward and reverse primer** | **Fragment length** | |
| *Hox1* | 5’- GAGCAAATGGACACGGCAAG -3’  5’- CTTCGACGGGCTATCTTCAC -3’ | 855 bp | |
| *Hox3* | 5’- GAGGTGGTGGCAGCTATGG -3’  5’- CGCAGTAGTTCATATTGACCAC -3’ | 1077 bp | |
| *Meis* | 5’- CAGTCGCCACGTCTATGTACG -3’  5’- CTGGACTATCCTGCGCCTC -3’ | 983 bp | |
| *Cyp26.1* | 5’- GCTGGTACTGGTGCTGTGGAG -3’  5’- ACATCACGCGCCGCTAACAC -3’ | 759 bp | |
| *Cyp26.2* | 5’- CTGCTGCTGTCCTGGAAGCTG -3’  5’- GTCTCCACTGTCTCCTCCCTG -3’ | 720 bp | |
| *Cyp26.3* | 5’- CAGACTTCTCCCGTAAGCGAC -3’  5’- CCCCAACAGGTGGACTTAGC -3’ | 891 bp | |
| *Cdx* | 5’- AAGACGAGGACGAAGGATAA -3’  5’- ACTGACCAGAGCCCTTTCCT -3’ | 192 bp | |
| *Pitx* (gift Z. Kozmik) | 5’- GCTTGGACCAACCTCACAGAG -3’  5’- TCGACGAACTCTGAACAGCT -3’ | 962 bp | |
| *Six1/2* | 5’- GTTCACCCAGGAGCAGGTCG -3’  5’- GTTACTTACGGCCACGGCG -3’ | 775 bp | |
| *IrxC* | 5’- GTCCTACCCACACTTTGGATAC -3’  5’- CCAGTGGCGGAGGTTAGCTAC -3’ | 1096 bp | |
| *NosC* | 5’- TCGGCCGAACGTAATTGCCG -3’  5’- GCCCGCATGAAGAACTGGCTG -3’ | 787 bp | |
| **Primers for quantitative RT-PCR** | | | |
| *Adh3* | 5’- GTCCCACAGTGCAAGGAGTG -3’  5’- CCACCGTGTACTCGCTGAAG -3’ | | 175 bp |
| *Rdh11/12_18* | 5’- CAGCAGGAGGGAAGTGTGAG -3’  5’- GGACGCAAGGTCAAGTTTCTG -3’ | | 227 bp |
| *Aldh1a_2* | 5’- GTAAGATCATCCAGGCAGCAG -3’  5’- CGTCGTAGACAGATTCCTCCAC -3’ | | 201 bp |
| *Crabp* | 5’- GTCAGCTTCAAGATCGGAGAG -3’  5’- CTTCATCACCAGGTACATCCG -3’ | | 171 bp |
| *RAR* | 5’- GTCGTCTGGCTACCACTACGG -3’  5’- ACCTGCAGAACTGGCATCTG -3’ | | 152 bp |
| *Hox1* | 5’- GGATACATGCACCACCATACG -3’  5’- GTCCGTCCGTTGTTGGGTCCG -3’ | | 176 bp |
| *Hox3* | 5’- CCGACAACAACCACAGCAG -3’  5’- CACAGGTAGCGGTTGAAGTGG -3’ | | 256 bp |
| *Meis* | 5’- CAGTCGCCACGTCTATGTACG -3’  5’- GAAAGAGTGGATGCCCGTAG -3’ | | 198 bp |
| *Cyp26.1* | 5’- GCTGGTACTGGTGCTGTGGAG -3’  5’- CGTGGAGAATCTTGCGCAC -3’ | | 248 bp |
| *Cyp26.2* | 5’- CAGGGAGGAGACAGTGGAGAC -3’  5’- CTTCTCCAGGTCCTCATGCAC -3’ | | 210 bp |
| *Cyp26.3* | 5’- CAGGAAGTTGCGGCATATCTTG -3’  5’- GTCGCTTACGGGAGAAGTCTG -3’ | | 190 bp |
| *Dmrt* | 5’-CTGGGTCTCCTGTACGGTAGTC -3’  5’- GTCCTCCTGTACCTTTCCCG -3’ | | 243 BP |
| *FoxE* | 5’- GACGGCTCAGCAGAATACAAG -3’  5’- GTTGTGTCGGATGGAGTTCTGC -3’ | | 214 bp |
| *RunX* | 5’- GATTCAACGACCTGCGCTTC -3’  5’- CTCTTCTAGCTTCTGCCGGTG -3’ | | 167 bp |
| *Pdvegfr* | 5’- CCAAAGGTGACCACCAACAGTC -3’  5’- GTCATTCTGGATGATGCGGC -3’ | | 150 bp |
| *Six1/2* | 5’- GCAGCTGAGGCGAAAGAGAG -3’  5’- CGCCGTGGCCGTAAGTAAC -3’ | | 258 bp |
| *Cdx* | 5’- GGCCCTGATGGTAAGACGAG -3’  5’- CTTCCGCTTGGCCATCTTG -3’ | | 213 bp |
| *Pitx* | 5’- GCTCACCGCCAAGTCGTTC -3’  5’- CTGGAATTGCACTGCTCACG -3’ | | 246 bp |
| *NosA* | 5’- AGTACAGTCATCTCCAGAAC -3’  5’- TCTTGCAAGCGCTTCTATCTG -3’ | | 221 bp |
| *NosB* | 5’- AGTTTACTCCCGGCGATCA -3’  5’- AGAACATGGCGGCAAACGC -3’ | | 191 bp |
| *NosC* | 5’- CAGGATTCTGCGCGTTTGC -3’  5’- GGAGCTAGCCTCGCTCATG -3’ | | 197 bp |
| *Rpl32* | 5’- GGCTTCAAGAAATTCCTCGTC -3’  5’- GATGAGTTTCCTCTTGCGCGA -3’ | | 117 bp |
